# The Application of Mendelian Randomization in Cardiovascular Disease Risk Prediction: Current Status and Future Prospects

**DOI:** 10.31083/j.rcm2507262

**Published:** 2024-07-11

**Authors:** Yi-Jing Jin, Xing-Yuan Wu, Zhuo-Yu An

**Affiliations:** ^1^Peking University Health Science Center, 100191 Beijing, China; ^2^Department of Cardiology, Peking University First Hospital, 100034 Beijing, China; ^3^Peking University Institute of Hematology, Peking University People's Hospital, 100044 Beijing, China

**Keywords:** Mendelian randomization, cardiovascular diseases, prediction model

## Abstract

Cardiovascular disease (CVD), a leading cause of death and disability worldwide, and is associated with a wide range of risk factors, and genetically associated conditions. While many CVDs are preventable and early detection alongside treatment can significantly mitigate complication risks, current prediction models for CVDs need enhancements for better accuracy. Mendelian randomization (MR) offers a novel approach for estimating the causal relationship between exposure and outcome by using genetic variation in quasi-experimental data. This method minimizes the impact of confounding variables by leveraging the random allocation of genes during gamete formation, thereby facilitating the integration of new predictors into risk prediction models to refine the accuracy of prediction. In this review, we delve into the theory behind MR, as well as the strengths, applications, and limitations behind this emerging technology. A particular focus will be placed on MR application to CVD, and integration into CVD prediction frameworks. We conclude by discussing the inclusion of various populations and by offering insights into potential areas for future research and refinement.

## 1. Introduction

Cardiovascular diseases (CVDs) are diseases involving the heart or blood vessels, including coronary heart disease, cerebrovascular disease, peripheral arterial disease, congenital heart disease, and aortic disease. As a non-communicable disease (NCD), CVD has been the leading cause of death and disability globally since the late 20th century [1], with a 2021 WHO report indicating they were responsible for 32% of all global deaths in 2019 [2]. The risk factors for CVD are diverse, ranging from lifestyle choices (including high-salt and high-fat diets, physical inactivity, smoking, and alcohol abuse) to medical conditions like hypertension, diabetes, dyslipidemia, and advancing age [3]. However, most CVDs can be prevented through a healthy lifestyle, while the early detection and treatment can significantly reduce the risk of severe outcomes [3]. With a global rise in the elderly population, there is an urgent need for comprehensive public health strategies and CVD-specific interventions to mitigate their worldwide health impact.

Mendelian randomization (MR) leverages genetic variations to estimate causal relationships between exposure and outcome with in quasi-experimental data, spanning observational studies such as cross-sectional, cohort, and case-control studies [4]. This approach identifies genetic variants, typically single nucleotide polymorphisms (SNPs), associated with an exposure but not with other risk factors or the outcome itself [5]. This isolates the relationship between the variant and the exposure, simplifying the process of inferring causality while minimizing confounding biases and reverse causality issues [6]. In recent years, MR has been widely used following genome-wide association studies (GWAS) and categorized into single-sample MR and two-sample MR depending on the number of datasets utilized. For two–sample MR, the instrumental variables (IV) exposure is estimated in the first dataset with outcome being estimated in the second dataset, whereas single-sample MR evaluates both relationships in a single dataset [7, 8]. Additionally, MR can be categorized as cis-MR, targeting variants from a single gene region biologically linked to the exposure, or polygenic MR which use genetic variants from multiple gene regions [8]. The choice between cis-MR and polygenic MR depends on the nature of the exposures [9]. Most often cis-MR is used when specific biomarkers including mRNA and proteins have been identified, whereas polygenic MR is applied when the exposure consists of complex multifactorial traits such as blood pressure and body mass index (BMI) [9]. In the last decade, MR has evolved into a valuable and proven method to elucidate the causal relationship between biomarkers and various diseases with CVD, while also identifying novel therapeutic targets [10, 11, 12].

Efforts to prevent CVD require early identification of individuals at higher risk, facilitating targeted interventions spanning diet, lifestyle, and pharmacotherapy. Over the past decades, numerous prediction models have been developed to gauge CVD risk by integrating multiple risk factors including the Framingham [13], SCORE, SCORE2 [13, 14], and QRISK [15] models. Key predictors in these models often include factors such as smoking, age, and sex [16]. Despite the surge in predictive models for CVD risk in recent years, challenges persist, including the lack of systematic descriptions of predicted outcomes, the integration of novel predictors, suboptimal predictive accuracy, and the need for external validation. In summary, MR plays a critical role in identifying novel metrics suitable for predictive modeling, while the development of comprehensive and effective CVD prediction models depends on incorporating these new metrics.

## 2. Mendelian Randomization: Principles and Methods

Epidemiology research on the link between exposure and disease has many limitations, as observational studies typically reveal associations rather than causality and are hindered by confounding variables, reverse causality, selection bias and many other factors. MR utilizes the link between genetic variation and exposure as an IV, facilitating the establishment of causal inferences. This is achieved by implementing randomization schemes within observational studies. MR uses exposure-related genetic variants as IV to make causal inferences by introducing randomization schemes into observational studies. As a result, MR is becoming increasingly popular for inferring risk factors for diseases, identifying biologic drug targets, and causal effects of genes on phenotypes [17, 18]. Therefore, careful selection of appropriate genetic variants is the most crucial aspect of MR research. Genetic variants that fulfill the following conditions are referred to as IVs [19]: (1) the genetic variant is associated with the exposure; (2) the genetic variant is not associated with any confounders of the exposure-outcome association; and (3) the genetic variant does not affect the outcome, except through association with the exposure. Only the first condition can be measured directly, while the other two can only be assessed through sensitivity analysis [20].

The use of IVs in MR enables the identification of associations between exposure and outcome that are free from confounding variables. This process mirrors the randomization process found in randomized controlled trials (RCTs), the gold standard for establishing causality. In MR, genetic variants create subgroups within the population, where exposure factors vary but are not randomly assigned. However, across a broad population, confounders including social and environmental factors are assumed to distribute randomly among these genetic variants, akin to the random assignment in RCTs [18]. This resemblance has led to MR being likened to “natural randomized trials” due to the equitable distribution of most genetic variants across populations [21].

Despite their similarities, MR and RCT possess distinct conceptual differences. (1) Purpose: MR is aims to determine if there is a causal relationship between the effect of an exposure and an outcome, while RCTs evaluate the clinical significance of the causal effect [4]. (2) Nature of the intervention: IVs in MR typically exert smaller, lifelong effects on exposures compared to RCT interventions [21]. Interventions in RCTs typically exert a greater quantitative impact on intermediate biomarkers influencing outcomes [21]. These interventions are characterized by shorter durations and are specifically designed for clinical diseases, pharmacologic interventions, and the prevention of relapse events [22]. RCT, on the other hand, requires prospective cohort studies with new interventions, which cost more money, labor, and time to complete while increasing precision [22].

In MR, monotonicity and homogeneity are key assumptions. Monotonicity ensures that the genetic variants used as IVs yield exposure effects that are consistent among the study subjects, supporting a uniform influence of exposure on outcomes [23]. These rules out possibility of IVs having divergent effects on the exposure, thereby simplifying the causal inference [23]. However, the presence of pleiotropic effects, where genetic variants impact multiple phenotypes, introduces complexity to MR analysis [24]. Homogeneity refers to the assumption that the effect of the genetic variants on the outcome, mediated through the exposure, is consistent across all individuals in the study. Variability in this area could arise from interaction between the genetic variants and other variables, such as age, sex, or lifestyle factors, that also influence the outcome.

Understanding the relationship between genetic variants, exposures, and CVD requires acknowledging the potential for non-linear dynamics. Traditional MR analyses often assume linear relationships between exposures and health outcomes. The introduction of non-linear analysis may be necessary when the data fail to meet the underlying statistical assumptions [25]. For example, the risk associated with blood pressure or lipid levels may not increase uniformly across their entire range [26]. Identifying points where risk increases or decreases more sharply can inform targeted interventions and refine risk stratification models [25]. Non-linear MR analysis has emerged as acritical tool in these scenarios, allowing researchers to identify thresholds or inflection points where the relationship between exposure and risk change, enhancing the accuracy of risk prediction models. This nuanced approach underscores the importance of considering the full spectrum of possible relationships in genetic epidemiology research, particularly for complex diseases like CVD.

The initial phase in MR involves identification of the study population, which can either be a single, large cohort with measurable data on exposures, outcomes, and genetic variant for each participant, or a comparative analysis of aggregated data from a GWAS between independent populations [27, 28]. The power of MR analyses increases proportionally to sample size when using individual-level data; when using pooled data, the precision of MR estimates depends on the precision of the association between genetic variants and outcomes [27, 28]. To improve precision, we can use efficacy calculations to determine whether sample sizes are adequate; simulation studies used to determine efficacy are also applicable to data with partially unique characteristics [27, 28]. The following phase involves the identification of IV, which can be either a single genetic variant with a robust association to the target exposure, or a composite genetic score comprising multiple independently associated variants with weaker links to the target exposure [29]. These genetic markers are typically SNPs derived from GWAS exploring the association between the SNP and phenotype using whole genome sequencing data from large populations [30]. These individuals were then aligned by defining the allele associated with the exposure level as the “exposure allele” for each individual in the variant [31]. Early MR used fewer genetic variants which were associated with risk factors, as IV to assess causal effect between exposure and outcome [32]. However, the limited sample size and number of IVs, providing insufficient statistical power, may have led to false-negative results. This is addressed through the use of single- or two-sample MR methods [19]. Single-sample MR requires a sufficiently large data set to assess all three variables: genetic variant genotype, exposure (risk factor), and outcome (disease) and uses two-stage least-squares (2SLS) regression to evaluate the causality [33]. With the robustness and increasing popularity of GWAS, two–sample MR has gained widespread adoption, allowing researchers to obtain genotype-exposure associations, and genotype-outcome associations across different samples, with the assumption of uniformity between genetic associations and their paired exposures between samples [34]. Ultimately, by measuring the associations between genetic variants and the target outcome, MR facilitates the conclusion that genetic variants associated with higher or lower levels of exposure are causally linked to the target outcome. This methodological advancement in MR, particularly with the introduction of two-sample MR leveraging GWAS data, has significantly enhanced the capacity to infer causal relationships in epidemiological research.

Importantly, while MR is rooted in causal inference, its application extends beyond to inform predictive modeling. The distinction between causal modeling, aimed at understanding the influence of exposures on outcomes, and predictive modeling, focused on forecasting outcomes based on a set of variables, is fundamental [35]. Yet, MR offers a unique bridge between these paradigms by providing scientifically rigorous, causally informed predictors for risk prediction models [36]. This approach not only enhances the accuracy and clinical utility of such models but also emphasizes the innovative role of MR in addressing confounding and reverse causation challenges in epidemiological research [37, 38]. Thus, our manuscript aims to elucidate the synergistic integration of MR-derived insights into CVD risk prediction, marking a novel contribution to the field.

## 3. Mendelian Randomization in CVD Risk Prediction

As CVD remains a leading cause of global mortality, development of more sophisticated risk prediction models have become imperative. Traditional MR has been utilized to assess causal inference, and has emerged as a powerful resource in this context [4]. By leveraging genetic variants as IVs, MR facilitates the identification of causal risk factors for CVD, thereby offering a robust foundation for enhancing predictive models [39]. This manuscript highlights the practical integration of causal insights gleaned from MR into the development of more nuanced CVD risk prediction models [40]. In accomplishing this, it bridges a critical gap in current epidemiological research, integrating causal understanding with predictive accuracy [40, 41]. There have been many studies applying MR to the development and improvement of CVD risk prediction models, covering a wide range of diseases such as coronary heart disease, heart failure (HF), and atrial fibrillation (AF) [39, 42, 43].

The utility of MR in constructing CVD risk prediction models is contingent upon the ability to navigate confounding variables and adhere to several critical assumptions [34, 44]. These assumptions are essential for ensuring the interpretability and validity of MR derived estimates, thereby enabling their effective incorporation into risk prediction frameworks [34, 44]. We delineate these assumptions below and discuss their implications for the development of accurate and applicable CVD risk prediction models [34, 44]. Relevance: the genetic variants employed as IVs must have a strong association with the exposure [34, 44]. This condition is vital to ensure that IVs sufficiently influence the exposure, allowing for a meaningful analysis of its impact on the outcome [34, 44]. Independence: the IVs should not be associated with confounders in the exposure-outcome link. Adherence to this assumption is critical for MR’s resistance to confounding variables, ensuring that the relationships observed are not biased by hidden variables [34, 44]. Exclusion restriction: the IVs must influence the outcome exclusively through the exposure without any alternate routes [34, 44]. Adhering to this assumption guarantees that the estimated causal effect accurately mirrors the true influence of the exposure on the outcome [34, 44]. By meticulously observing these assumptions, MR can significantly enhance the development of CVD risk prediction models, offering a pathway to better understand and predict cardiovascular risk with greater accuracy and applicability.

### 3.1 Risk Factor Identification

The prevention of CVD hinges on the identification and management of common risk factors including obesity, hypertension, hyperglycemia, and hyperlipidemia, which are metabolic aberrations [45, 46, 47]. Li *et al*. [45] focused on basal metabolic rate (BMR) and its implications for CVD utilizing univariate MR analysis using GWAS data. Their findings revealed that genetically predicted higher BMR is linked to an increased risk of atrial fibrillation and heart failure, yet conversely decreases the risk of myocardial infarction [45]. This underscores BMR’s nuanced role in different CVD outcomes and highlights its importance in the context of human aging and CVDs. Furthermore, a large-scale GWAS identified 102 new loci associated with visceral adipose tissue [46]. Utilizing MR analysis, this study demonstrated that visceral fat acts as a causative risk factor for hypertension, angina, type 2 diabetes, and hyperlipidemia [46]. The development of gender-stratified nonlinear prediction models based on these findings indicated a disproportionately higher causative risk for hypertension and type 2 diabetes in females, which may be related to the higher average mass of visceral fat in females [46]. In a prospective study of Chinese coronary artery disease (CAD) patients [47], MR analysis identified 11 genetically inferred metabolic profiles that were associated with adverse cardiovascular events and left ventricular remodeling. Importantly, a predictive model integrating four specific metabolites was able to successfully identify patients at high risk of death and major adverse cardiac events (MACE) in a multicenter validation cohort [47]. This achievement marks a significant step forward in enhancing risk stratification for CAD patients, suggesting that metabolic alterations seem to contribute to MACE by promoting left ventricular dysfunction, supporting some of the potential therapeutic targets.

The significance of classical risk factors in the elderly remains unclear. An MR study [43] evaluated the causal relationship between selected classical risk factors (sex, BMI, blood pressure, low-density lipoprotein cholesterol (LDL-C), triglycerides) and primary CAD in different age groups in a European population to assess the predictive power of genetic risk scores with age. The researchers observed that with increasing age at diagnosis, genetically influenced CVD risk factors progressively reduced the risk of primary CAD [43]. This relationship was more pronounced in women aged over 60 [43]. These results suggested that the age-based dynamics of CVD risk factors should be considered when evaluating the association between risk factors and diseases by MR. Lipid-lowering recommendations for the prevention of CAD rely heavily on the prediction of risk over a 10–year period [48]. Pencina *et al*. [48] used both RCT and MR predictive models to estimate the absolute and relative effects of age and non-high-density lipoprotein cholesterol (non-HDL-C) levels on the expected incidence of CAD over the next 30 years in a relatively young patient population (30–59 years). The results indicated that in the middle-aged population, non-HDL-C ≥50 mg/dL increases the risk of CAD, while intensive lipid reduction at this point significantly reduced the expected CAD risk over the next 30 years [48]. The prediction improved with both advancing age and elevated non-HDL-C levels [48].

The integration of DNA methylation (DNAm) profiles into CVD prediction models represents an innovative and promising avenue of research. These studies have linked DNAm’s with various CVD risk factors including lipids, blood pressure, BMI, and postprandial lipids [49, 50]. Methylation-based risk scores (MRS) have already demonstrated their capability to predict myocardial infarction events in the Framingham dataset, notably offering improved predictions for individuals at lower risk, facilitating the identification of individuals overlooked by other models [51].

However, studies connecting DNA methylation and CVD face challenges like reverse causality, making MR an invaluable methodology for reinforcing the evidence base. Huan *et al*. [52] analyzed 15,013 samples from 15 prospective cohort studies, applying multi-IV MR analyses on three CpGs to explore their differential methylation’s causal relationship with CVD outcomes. Incorporating CpGs associated with CVD into a prediction model, alongside age, sex, and twelve additional clinical risk factors improved CVD mortality predictions by 2% [52]. This model showed superior predictive performance across four different DNAm age models.

### 3.2 Inflammation and CVD Risk

Furthermore, the role of inflammation in CVD progression cannot be overlooked. Aslibekyan *et al*. [53] examined 11,461 subjects with CAD, investigating associations between circulating levels of tumor necrosis factor α (TNF-α) and whole-blood DNA methylation. They discovered a strong negative correlation at all four TNF-α–related sites, with a 10% increase in methylation at these sites correlating with a 9%–19% reduced risk of adverse coronary events [53]. This finding solidifies the significance of TNF-α methylation as a potent biomarker for assessing CVD risk. Additionally, mitochondrial DNA (mtDNA) dysfunction during inflammation has been linked to CVD development. Studies of MR have explored the causal relationship between leukocyte mtDNA abundance and CAD and HF [54]. It was discovered that reduced leukocyte mtDNA may be causally related to an elevated CAD risk, a connection not observed with HF [54]. This body of work exemplifies the evolving landscape of CVD risk prediction, highlighting the critical role of genetic and epigenetic factors in advancing our understanding and management of cardiovascular health.

### 3.3 Alcohol and CVD Risk

The relationship between alcohol consumption and CVD remains a subject of debate, with studies indicating both harmful effects from heavy drinking and protective benefits from light to moderate consumption. This inconsistency is particularly pronounced across specific populations, complicated further by the influence of socioeconomic, lifestyle, and psychological factors that challenge precise characterization. Previous MRs were mostly conducted on European populations and often relying on summary-level data, failing to provide specific quantitative descriptions. The utility of MR lies in its use of genetic variation as a tool to mitigate racial genetic differences, enabling the development of predictive models for specific races, countries, and between sexes. This approach allows for a more nuanced exploration of alcohol consumption and CVD in a more in-depth and fundamental way.

Using a prospective Asian cohort, Hu *et al*. [55] found a linear increase in CVD incidence and mortality with rising alcohol intake, suggesting no genetic safe consumption threshold. Conversely, research within a Chinese population [56], explored the relationship between genotype-predicted mean alcohol consumption, carotid plaque, and carotid intima–media thickness (cIMT) across both sexes. These findings indicated a significant association between greater alcohol intake and increased carotid plaques in males—attributable primarily to alcohol rather than pleiotropic genetic effects—while no clear dose–response relationship emerged among females [55].

Similar to alcohol, the link between tea consumption and CVD is inconclusive. Multiple MR analyses [57] probing the habitual tea use-CVD association concluded no genetically predicted causal relationship, underscoring the complexities in determining the impacts of lifestyle factors like alcohol and tea consumption on cardiovascular health. These studies highlight the importance of considering genetic backgrounds and individual differences when assessing risk factors for CVD, providing a more comprehensive understanding of the interplay between genetics, lifestyle choices, and cardiovascular outcomes.

### 3.4 Diagnostic Criteria and CVD Risk

Diagnostic criteria for CVD include, blood biochemical tests in addition to clinical signs and symptoms. Notably, MR has been instrumental in the discovery of novel biomarkers. Observational studies have linked high circulating blood copper levels with increased CVD risk through a mechanism involving inflammation-induced oxidative stress [58, 59]. However, some MR studies have presented opposing results [60]. In particular, Jäger *et al*. [61] investigated the causal association of genetically related blood copper levels with stroke, CAD, and type 2 diabetes mellitus in a two-sample MR study. Their results demonstrated that elevated genetically-induced copper levels were inversely related to CAD and systolic blood pressure [61]. Furthermore, integrating blood copper levels with systolic blood pressure in a predictive model suggested that blood copper may influence CAD risk through systolic blood pressure modulation [61].

The causal relationship between apolipoprotein B (apoB) and CAD has been extensively studied, with recent studies have focused on the relationship between apoB and non-HDL-C/apoB particle concentrations [62]. Utilizing 235 variants as IVs for single-sample MR analysis revealed that both non-HDL-C and apoB were associated with the risk of CAD in a dose–dependent manner [62]. Importantly, incorporating non-HDL-C into a model already featuring apoB markedly enhanced the prediction of genetically determined CAD, a finding not reciprocated when apoB was added to a non-HDL-C inclusive model [62]. This pattern held true across five CAD datasets, suggesting that for individuals with similar non-HDL-C levels, the quantity of apoB particles does not influence CAD development [62]. Furthermore, the investigators focused on genetic variations associated with triglycerides, using non-HDL-C/apoB as secondary hypertriglyceridemia risk enabled the identification of 51 sequence variants [62]. This highlights the strong genetic contribution of apoB particles to cholesterol, which was particularly evident under statin–treatment, suggesting the clinical benefits of lipid–lowering therapies may derive from non-HDL-C reduction, rather than apoB levels [62].

Moreover, in a cohort from an Icelandic population [63], MR–predicted non-HDL-C levels exhibited a stronger association with CAD than LDL-C, supporting non-HDL-C as a superior biomarker for overall CAD lipoprotein burden. Additionally, Henry *et al*. [39] conducted an observational study on four independent samples, identifying 44 circulating associated with an increased risk of heart failure. Primary MR analysis confirmed that 17 of these proteins had a causal association with heart failure [39]. Interestingly, among proteins not correlated in the observational study, MR identified nine proteins were causally associated with heart failure [39]. Furthermore, primary cis-MR analysis was used to assess eight proteins closely related to heart failure, exploring the causal relationship with heart failure-related symptoms, aiding in identifying potentially novel drug targets [39]. In AF, a study [64] identified 91 new genetic loci by through a GWAS analysis, enhancing AF-related comorbidity predictions with a polygenic risk score. This breadth of research underscores the multifaceted approach to understanding CVD, leveraging genetic insights to refine risk assessment and unearth new therapeutic avenues.

### 3.5 Comorbid Diseases and CVD Risk

While exploring the relationships between CVD and disease comorbidities, Wang and Ding [65] utilized two-sample MR to demonstrate that genetic predisposition to major depression is linked to an elevated AF risk, highlighting the intersection between mental health and CVDs. While chronic kidney disease (CKD) was identified as an independent CVD risk factor, traditional cardiovascular risk prediction tools, derived from the general population, are less effective in CKD patients [66]. Through MR analysis focused on a non-dialysis-dependent CKD cohort, researchers identified 18 proteins causally associated with adverse CVD outcomes [66]. This led to the development of 32 protein models to predict the risk of myocardial infarction, heart failure, stroke, and cardiovascular death in this population, which is more applicable to proteomics modeling risk stratification than existing clinical risk models [66].

Additionally, the relationship between type 2 diabetes mellitus (T2D) and CAD was explored through the construction of a genetic risk score (GRS) based on T2D genetic variants [67]. The GRS’s association with the severity of CAD in patients with acute coronary syndromes (ACS) indicates a linear relationship between GRS and an increased risk of multivessel disease in ACS patients [67]. This suggests that the impact of T2D on CAD risk may be partially mediated through genetic predispositions, providing a clearer understanding of how T2D contributes to cardiovascular complications. These studies collectively emphasize the importance of genetic and proteomic analyses in uncovering the complex interplay between various diseases and their impact on cardiovascular health.

In summary, we can see the advantages that MR possesses in the prediction of CVD risk. (1) Targeted insights: for different genders, ages, ethnicities and other risk factors with a strong genetic relationship, MR can provide more accurate evidence of causality. This is particularly critical for CVDs, where genetic links play a significant role in disease development. (2) Stability: unlike traditional observational studies, which rely on questionnaires, biochemical markers, and imaging, genetic variation begins at birth and remains relatively stable throughout the lifespan. This constancy ensures that MR-derived associations are not subject to causal inversion and are minimally influenced by confounding factors, offering more reliable insights into causal relationships. (3) Simplicity and accessibility: MR leverages widely accessible GWAS data. Resources like the UK Biobanking Cohort [68], MR-BASE platform [69], and similar GWAS summary data provide extensive information on genetic instruments, human traits, diseases, and diverse population samples. Compared to the complexity and logistical challenges of RCTs, MR offers a straightforward and efficient approach to research, potentially conserving significant time, effort, and financial resources. (4) Timeliness: for many risk factors, traditional RCTs may not be able to assess long-term cardiovascular outcomes, such as smoking—MR studies are exceptionally suited for these contexts. They can evaluate the lifelong implications of exposures on disease risk, offering timely and relevant insights that might not be feasible to obtain through RCTs. Overall, MR’s unique strengths in targeting, stability, simplicity, and timeliness underscore its potential to enhance our understanding of CVD risk factors and to inform the development of effective prevention and treatment strategies.

## 4. Current Limitations and Future Directions

The inherent characteristics of genes provide a solid basis for their use as IVs in MR. However, there are still many limitations for applying MR analysis in CVD. For instance, alcohol research with MR is complex due to non-genetic factors like reporting honesty and educational background, which introduce confusion [70]. Alcohol-related genes have multiple genetic loci and the genetic loci can affect outcomes through many different pathways. This includes their effect on alcohol exposure levels in addition to alcohol metabolites which can influence health outcomes [71]. Such scenarios can violate the core assumptions of MR IV [71]. Furthermore, the applicability of MR’s instrumental variable assumptions and their biological plausibility might not always hold true [24, 72]. Genetic variation does not always adhere to Mendel’s law of independent assortment, with linkage disequilibrium illustrating that not all traits’ determining genes segregate independently and randomly [24, 72].

Violation of (1)–(3) of the aforementioned instrumental variable conditions introduces “weak instrument bias”, “uncorrelated horizontal pleiotropy (UHP)”, and “correlated horizontal pleiotropy (CHP)”, respectively [24, 72]. Horizontal pleiotropy (HP) occurs when a genetic variant influences the outcome through pathways other than the exposure [24, 72]. Meanwhile, CHP refers to situations where the correlation between an IV and both the exposure and outcome is generated from shared confounding variables affecting both systems [24, 72]. Finally, UHP occurs when an IV’s effect on the outcome is not related to its effect on exposure, meaning the instrumental variable impacts the exposure and outcome through two different mechanisms [24, 72].

The presence of these two levels of pleiotropy tends to result in higher false positives for MR. In practical applications, the availability of cis-eQTL (expression–quantification–trait–loci) for most genes in the genome that are used as IVs is limited, and frequently reflects the complex genetic basis of traits and pathways between the traits [73]. While there are solutions for both levels of pleiotropy, most are not well developed [74]. Notably, MR polytropic residuals (MR-PRESSO) [74], iterative MR polytrope (IMRP) [75] by hypothesis testing, and MR-Egger by regression analysis can address UHP. Weighted Median, MR-Robust, and MR-Lasso attempt to solve UHP/CHP problems by robust loss function [76]. Unlike UHP whose impact was appropriately addressed by most of the previous MR studies, some researchers may have ignored CHP, which would have resulted in a higher rate of false positives.

In recent years, some models have also been developed to be able to address the effects of both UHP and CHP at the same time [24, 77, 78, 79, 80], including Gaussian mixture models, MR contamination mixture (MR-Conmix), causal analysis using summary effect (CAUSE), MR constrained maximum likelihood (MR–CML), MR with correlated horizontal pleiotropy unraveling shared etiology and confounding (MR–CUE). The use of multiple genetic variants in combination in MR can improve statistical efficacy, as combinations are valuable for detecting or avoiding bias when certain genetic variants do not satisfy IV conditions [34]. When using SNPs, the overlap between the dataset used to select for genetic variation and the dataset used for measurement can lead to a “winner’s curse”, causing an overfitting bias. However, recent studies have demonstrated that these bias effects can be accounted for and corrected when strong instrument variables are used [81]. Through the use of an F statistic >10, the bias can be significantly decreased. Meanwhile, a “collider bias” occurs when both the exposure and the outcome affect a third variable, and that variable is controlled for in the study design [82]. When both exposure and outcome affect a third variable and that variable is controlled for in the study design, “collider bias” occurs, an outcome which is less easily observed [82]. Similarly, the IVs used in multivariable MR are a concatenation of the exposure-specific IVs used in univariate MR [83, 84]. As the GWAS sample size grows, more and more small and moderate causal variants are identified [83, 84]. The accumulation results in “weak instrument bias”, a phenomenon that can be addressed through proper calibration. This bias can be mitigated through the correction of weighted least squares equations [85, 86, 87, 88]. A multivariable MR approach, specifically the novel bias-corrected estimating equation (MRBEE) has been developed to estimate the causal effect of exposures with minimal bias and optimal frequency of coverage [89]. It effectively addresses the challenges posed by CHP, UHP, variations in GWAS sample sizes, and weak instrument bias [89]. Furthermore, this method was validated in the analysis of real-world CAD data [89].

Deciphering causal relationships directly from genomic data presents significant challenges. When genetic variation is significantly associated with the expression of a single gene, it allows for straightforward hypothesis formation and inference. However, when genetic variation is associated with multiple target genes, or when there is an inter-regulatory relationship between target genes, the application of causal network inference becomes important [90]. Several methods have been proposed to improve precision and efficiency. The multi-tissue dual-sample MR method ROBust to invalid IV (MR-MtRobin) [91], uses eQTL summary statistics. It identifies and corrects for errors due to pleiotropy, enabling accurate causal inference even in the presence of null IV [91]. Another method, MR-${\mathrm{Corr}}^{2}$, uses GWAS summary-level data to account for correlated HP and genetic variation. It efficiently models polygenic SNPs in linkage disequilibrium through a binary normal distribution approach, including an efficient algorithm with paralleled Gibbs sampling to infer the posterior mean of causal effect [80]. These advancements signify important progress towards more precise and efficient causal inference in genomic research, especially in the context of pleiotropy and genetic linkage.

The quality of analysis and reporting can vary greatly between MR studies. Despite genes serving as IV, they are still susceptible to biases from multiple factors including weak instruments, challenges in ensuring the multiplicity of validity, sample overlap, complications with back-text variants, difficulties in variant replications, missing data, associations of IV with both the exposure andoutcome, and bias introduced by one-sample and two-sample methods [92]. The use of MR in CVD has specific limitations.

### 4.1 Poor Generalizability

Significant heterogeneity exists among CVD-associated SNPs, and thus the results of MR need to be interpreted with caution. If the available study data are limited, the generalizability of expected results is often restricted to a particular region and ethnicity [42, 93]. These differences emphasize the need for further studies to elucidate underlying mechanisms and to understand the influence of genetic and environmental factors [42, 93]. At the same time, the associations identified may not directly reflect observations from animal-based mechanobiological studies [94]. One solution to this shortcoming would be to include data from as many different ethnic groups as possible, which may further introduce bias due to population stratification and admixture [95]. The acquisition of larger scale GWAS data remain an important goal for future studies.

### 4.2 Challenges of Time-Varying Exposures in MR

One inherent challenge in MR studies is the assumption that the effect of genetic variants on an exposure is constant throughout an individual’s life. The evolving nature of exposure over time has always been a major challenge for MR studies. Exposures may change over time, and their impact on disease risk can vary at different life stages. Certain exposures are only correlated with outcomes during specific periods. Richardson *et al*. [96] utilized Life Course MR to elucidate the relationship between body size at different stages of life and the risk of coronary heart disease (CHD). Life Course MR treats exposures at different time points as separate exposures for MR analysis, employing both univariable and multivariable MR to assess the direct and indirect effects of exposures at different time points on the outcome. The results show that while body size in early life is associated with CHD risk, multivariable MR suggests that the direct effect of adult body size predominates in influencing CHD risk. This result is also supported by real-world data studies. Despite one large clinical study indicating an increased risk of CHD in adulthood associated with higher BMI during childhood, subsequent research has demonstrated a significant reduction in CHD risk after weight loss surgery, suggesting a direct association between adult obesity and CHD [97, 98, 99]. O’Nunain *et al*. [100] also used a similar approach to demonstrate that body size during childhood, rather than adulthood, influences cardiac structure in adulthood. By examining the specific critical periods when exposures exert their effects, we can more accurately control exposure factors to reduce the risk of disease occurrence. This complexity is particularly relevant for CVD, where risk factors such as blood pressure and cholesterol levels may have different impacts depending on the age of the individual [101].

### 4.3 Potential Pitfalls in MR Interpretation

Estimates used in MR can be skewed by several factors, which if not properly addressed, can mislead interpretations. One significant challenge is pleiotropy, where genetic variants used as IVs affect multiple phenotypes beyond the exposure under investigation, potentially biasing causal estimates. Another issue is population stratification, which occurs when differences in allele frequencies and disease risks across various populations due to ancestry lead to false associations. Additionally, measurement errors in exposure assessment can also introduce biases into MR estimates. These inaccuracies in quantifying exposures can compound, further skewing the results and complicating the causal inference drawn from MR studies.

### 4.4 Challenge of Non-Linear Mendelian Randomization

As mentioned earlier, conventional MR methods assume a linear association between exposure and outcome, which may not always hold true. Staley and Burgess [26] were the first to use semiparametric methods, including the fractional polynomial method and a piecewise linear method, to estimate the nonlinear relationship between BMI and systolic and diastolic blood pressure. This approach was widely used in later CVD research, particularly in assessing the L-shaped relationship between vitamin D and CVD risk [25].

However, as research progressed, some began to question the validity of the assumption that genetic variation has the same effect on stratified exposures in these methods [102]. Burgess *et al*. [103] employed the doubly-ranked method to mitigate this problem, and found the original results no longer held after using the updated method, indicating significant flaws in traditional nonlinear MR. Wade *et al*. [104] further refuted the validity of existing nonlinear MR through negative control methodology. They found that both semiparametric methods and the doubly-ranked method resulted in a higher likelihood of participants who have lower BMI to be female, even though BMI cannot affect gender [104]. This suggests that caution should be used when approaching and interpreting the conclusions of existing nonlinear MR studies.

### 4.5 Difficult to Assess Qulity

Due to the growth in the number of MR studies, the need for standardized methodology guidance has become apparent. Reviews of the MR literature have identified a broad spectrum of issues across numerous studies, leading to questions about the quality and reliability of their findings [92, 105]. In response to this, the Reporting of Observational Studies Using MR to Enhance Epidemiology (STROBE–MR) guidelines were developed [106]. These guidelines detail key objectives for various aspects of MR research, including the background, purpose, study design, data sources, statistical methods, and sensitivity analyses [106]. They fulfill a critical role by helping researchers navigate methodological decisions effectively throughout their studies and by providing a basis for evaluating the quality, limitations, and findings of MR research. This framework is vital for ensuring the methodological integrity and relevance of MR studies, thereby improving their generalizability and the usefulness of their conclusions in broader epidemiological contexts.

### 4.6 Poor Precision

The interpretability of effect estimates in MR studies is constrained, as they do not directly indicate the predicted change per unit of change in the exposure phenotype [107, 108]. Additionally, determining the linearity relationship often requires larger studies for confirmation, while accessing individual patient-level data presents significant challenges [94, 95]. Variability in data sources introduces distinct potential biases [109], which can be somewhat controlled through adjustments for pooled associations using covariates. Employing larger datasets that include individual-level data could elucidate more precise relationships such as dose and threshold effects [110]. A notable gap in current MR research is the lack of studies conducted during disease progression, which typically have a latency period [111]. Biobanks linking participant data to electronic health records offer a promising avenue for gaining insights into disease progression [111].

Finally, as MR studies rely on genes as IV, it is imperative to emphasize several critical regulatory and ethical considerations. It’s essential to have stringent procedures for obtaining informed consent from study participants, ensuring participants fully understand the genetic nature of the research and its potential implications. Protecting against genetic discrimination is paramount, necessitating measures to safeguard individuals from any negative impacts stemming from analysis of their genetic data. Additionally, transparency and interpretability in MR research are also vital aspects that warrant thorough attention. Researchers must strive for clear communication of methodologies, results, and potential limitations to ensure the integrity and comprehensibility of their work. Addressing these ethical, regulatory, and methodological issues is imperative for advancing MR research and maximizing its contributions to understanding complex diseases.

## 5. Future Applications of Mendelian Randomization in CVD Diseases

Recently MR has evolved from an experimental technique to a versatile tool with applications extending beyond the study of diseases and their risk factors. Nowadays, researchers utilize MR in varied contexts, tailoring the choice of exposure, outcome, or IV to suit the specific problem at hand. This flexibility allows for the exploration of complex relationships across a broad spectrum of disciplines, ranging from environmental science to behavioral genetics and beyond. By selecting different types of exposures, outcomes, or IVs, researchers can adapt MR to address diverse questions, providing valuable insights into the causal mechanisms underlying various phenomena.

### 5.1 Drug Target Identification

The increasing use of MR has led to its adoption for the identification of potential targets for novel pharmaceutical treatments. Prior to the integration of MR into this investigative process, GWAS successfully identified proprotein convertase subtilisin/kexin type 9 (PCSK9) and angiopoietin-like 4 (ANGPTL4), two prominent targets for lipid-reduction therapies [30]. Compared to traditional single sample or two-sample MR, the introduction of IVs represents a watershed moment in the field. Unlike traditional approaches that employed SNPs as the IV, contemporary MR utilizes genetic variants that are directly linked to the function or expression of the drug targeting protein [112, 113]. When using the expression level of certain protein or mRNA as the trait, the genetic variation identified by GWAS will become quantitative trait locus (QTL). MR studies dedicated to drug target identification leverage these QTLs as IVs, with a specific disease as the outcome, facilitating the exploration of new drugs applications [112, 113].

Treatment of CVD, with the prevalence of chronic and degenerative conditions, requires careful identification and selection of drug targets. Aortic aneurysms (AA) represent a significant life-threatening condition without any satisfactory therapeutic interventions to decelerate clinical progression. Utilizing MR analysis, Chen *et al*. [114] highlighted four potential drug targets for AA treatment, specifically proteasome 20S subunit alpha 4 (PSMA4), plasminogen activator, and urokinase. Further studies suggested that inhibiting plasminogen activator, urokinase (PLAU) and PSMA4 could mitigate AA risk without exacerbating cardiovascular or metabolic diseases. Expanding the scope to aortic stenosis, another formidable cardiovascular challenge, Ciofani *et al*. [115] evaluated the potential of immunomodulatory drugs through MR. Instead of using traditional QTL as IVs, the research team used genetic proxies, specifically SNPs associated with serum CRP levels to identify drug target proteins [115]. The study identified tocilizumab, an interleukin 6 (IL-6) inhibitor, as a potential therapeutic target [115].

While drug target MR studies are valuable for assessing outcome traits, gaining deeper insights into drug efficacy, adverse reactions, and potential repurposing opportunities, the technique is not without limitations. For instance, it cannot account for post-transcriptional and post-translational modifications, which can significantly influence the activity and function of proteins targeted by drugs. Additionally, drug target MR may not fully capture the variations in drug effects across different tissues and populations, highlighting an essential aspect of pharmacodynamics and pharmacokinetics that require either preclinical or clinical studies.

### 5.2 Determining Ancestry–Specific Causal Relationships

The migration and colonization patterns of human civilization have created a tapestry of genetic diversity across different sociocultural and ethnic groups, leading to significant variations in allele frequencies [95]. This genetic heterogeneity has imparted unique CVD epidemiological patterns across different populations. Understanding the population specificity of risk factors is crucial for the development of precise health care strategies. In this context, MR analysis, which relies on the genome data from large populations, underscores the critical role of careful population selection. Initially, MR research predominantly focused on European populations driven by the early establishment of comprehensive databases, such as the UK biobanks and FinnGen in Europe. However, the advent of databases encompassing a broader spectrum of genomic data and phenotypic details from a variety of ethnic groups has expanded the utility of MR. It has evolved into a powerful tool for identifying ancestry–specific causal relationships, enabling researchers to tailor healthcare interventions more accurately to the genetic and epidemiological profiles of different populations.

Several studies have employed MR to compare the causal relationship of risk factors and CVD between European and East Asian populations. Particularly, Wang *et al*. [116] reported that the risk factors of CAD largely overlap between these two populations, with the notable exception of uric acid and BMI, which have a greater impact on CAD risk in East Asians. The similarity of causal relationships was supported by Ciofani *et al*. [117], who assessed the link between traditional CVD risk factors and disease outcomes. However, they noted a distinct pattern in Europeans, where ischemic stroke and heart failure were significantly associated with all risk factors, whereas in East Asian populations only elevated blood pressure was significantly associated with these conditions [117].

While MR can be instrumental in uncovering ancestry–specific causal relationships, similar to findings from meta-analyses, challenges persist. These include the small sample sizes of genomic data from non-European populations and potential selection bias [118]. It is also essential to consider that non-causal risk factors may serve as proxies for other difficult-to-measure causal factors. Their inclusion in risk prediction models can offer valuable insights into the complex interplay of genetic and environmental factors contributing to CVD. Future research should aim to integrate both causal and non-causal factors to refine and enrich the precision and accuracy of CVD risk prediction models, thereby fostering a more nuanced understanding of disease mechanisms across diverse populations.

## 6. Conclusions

MR has emerged as a transformative tool in CVD risk prediction, leveraging genetic variation to elucidate causal relationships between risk factors and disease outcomes. Despite its potential, MR faces challenges such as pleiotropy and population stratification, which can affect the generalizability of findings across different ethnic groups. Looking forward, MR promises to refine CVD risk models through advanced analytics and expansive genomic datasets, offering insights into genetic influences on CVD and unveiling new targets for prevention and treatment. As we navigate its complexities and ethical considerations, MR stands as a beacon in genetic epidemiology, poised to enhance our understanding and management of CVD.
